# Computational science: shifting the focus from tools to models

**DOI:** 10.12688/f1000research.3978.2

**Published:** 2014-06-17

**Authors:** Konrad Hinsen

**Affiliations:** 1 Centre de Biophysique Moléculaire (UPR4301 CNRS), Rue Charles Sadron, 45071 Orléans, France; 2 Synchrotron SOLEIL, Division Expériences, St Aubin, 91192 Gif sur Yvette, France

## Abstract

Computational techniques have revolutionized many aspects of scientific research over the last few decades. Experimentalists use computation for data analysis, processing ever bigger data sets. Theoreticians compute predictions from ever more complex models. However, traditional articles do not permit the publication of big data sets or complex models. As a consequence, these crucial pieces of information no longer enter the scientific record. Moreover, they have become prisoners of scientific software: many models exist only as software implementations, and the data are often stored in proprietary formats defined by the software. In this article, I argue that this emphasis on software tools over models and data is detrimental to science in the long term, and I propose a means by which this can be reversed.

## Introduction

Computers have become an essential tool in many aspects of science: they help with collecting and processing data from observations, evaluating theoretical models, and communicating with fellow scientists. In the course of the few decades in which scientists have used computers, computing technology has changed very rapidly. These changes have permitted significant progress in many fields of science. However, they have also lead to a shift of focus from scientific to technological issues, as scientists eagerly applied new computing technology to study ever more complex systems. The most visible consequence is that today high performance often takes priority over reliable results in computational science, even though few scientists would openly admit this preference.

Recently, some of the negative consequences of rushing forward at a fast pace have become too visible to be ignored ^1– 3^: mistakes due to insufficiently verified software, lack of reproducibility due to incomplete publication of data and codes and blind trust in software without a deeper understanding of the methods applied. The frenzy of becoming ever faster is slowly giving way to a more sober attitude that reinstates reliability and verifiability as the prime values of science. The Reproducible Research movement ^4^ argues that reproducibility, one of the core principles of science, must be required in computational science as well as in other sciences, necessitating the publication of all software and data sets that are used in a computational study. From a somewhat different angle, the Open Science movement ^5^, whose goal is access for everyone to the process of research, comes to the same conclusion. As a result of these efforts, publishing scientific data and software has become not only possible but straightforward, and journals are starting to encourage or even require such publication to accompany the traditional article that describes a study’s methods and results.

If the code and the input data of a computational study are published, anyone could repeat the computation and verify that it produces the published results. This is often referred to as *replicability*. At this time there is no agreement on whether replicability is a useful characteristic of a scientific study; the references 6 and 7 show two opposing points of view. Replicability limits fraud by proving that the authors can actually compute the results that they show in an article. It can also be seen as a proof of quality assurance, because it demonstrates that the authors have recorded their complete computational workflow, which is not yet common practice. However, replicability does not mean that the authors did what they describe in their article, nor does it help the readers to develop a better understanding of the methods that were applied. Minimal replicability (such as making available a virtual machine image that runs the computation) doesn’t imply openness either, as readers cannot apply the published methods to different situations, or analyze the data using their own methods.

Replicability is clearly not the same as the traditional notion of reproducibility in science. The latter requires that other scientists design *their own* experiments or computations, which incorporate the key elements of the original work but differ in points considered unimportant, and obtain similar results. In contrast to replication, reproduction of a scientific study adds new information that helps to identify what matters and what doesn’t matter for obtaining a specific result. Making a computational study reproducible thus requires explaining the methods behind it in a way that clearly states which aspects are considered important.

The central question in computational science is: why should we trust the results of a non-trivial computation? We all know from experience that software has bugs, and we also know that the use of computers is subject to frequent human mistakes. Moreover, computational scientists should be aware of the complexity of their software, and thus should be concerned that it might not do what they believe it does. Creating trust in computational results requires validation at all possible levels: we need replicability *and* reproducibility, and also an increased effort to explain our computational models and methods to our peers. The fundamental problem is that scientific software is much too complicated to be an efficient way to communicate these models and methods, and no other precise representation is available. A detailed understanding of what a given piece of software does is often limited to the software’s authors.

In this article I will explain why the current situation is unsatisfactory, and propose approaches for improving it. I will illustrate my explanations with examples from my own field of research, which is biomolecular simulation. However, after many discussions with computational scientists from other application domains, I conclude that the situation is very similar wherever computers are used for tasks that are impossible to do manually.

In order to make my point clear, I will first give a summary of the role of models in science, and of the role of computation in scientific models. This will set the stage for the following discussion on the current state of scientific software. I will then propose concrete actions that can be taken to improve the situation and outline the benefits that we can expect from them.

## Models

**Figure 1.**
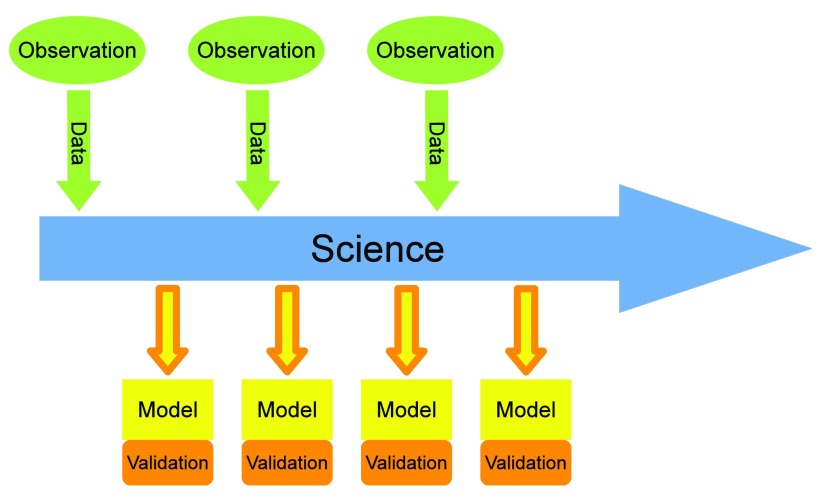
A scheme of the process of scientific research. The main input is data from observations, the main output are models with associated domains of validation.

The central concept presented in this article is the notion of the *scientific model*. The role of models in science has been the subject of much debate among philosophers of science ^8^. A good general overview written by a scientist for scientists ^9^ and an illustration in the context of physics education ^10^ have been given by Hestenes. A scheme of the process of scientific research (see Figure 1) illustrates the fundamental role of scientific models: science can be summarized as a process whose inputs are the data obtained from scientific observations, and whose output is a set of models with the associated validation information. As more data become available, new model/validation pairs are produced, which may be refinements of older models, but also completely new models. The defining characteristic of a scientific model is that it can be used to deduce verifiable statements about observable aspects of nature, which makes it possible to test and refine a model using new data from subsequent observations.

Models do not necessarily need to be quantitative. The metabolic pathways in biochemistry are a well-known example for non-quantitative models. However, in the context of computational science, nearly all models are quantitative, as they predict numbers that are compared to the numbers obtained from the actual measurements. In the following, I will limit the discussion to quantitative models.

The models most frequently discussed in the context of scientific research are those for the systems in nature that we try to understand. However, we also use physical models to describe the instruments we use to make observations, and non-physical phenomenological models to account for the aspects that we do not understand in detail. The most common models in the last category are the statistical error models, such as the very frequently (and usually silently) made assumption that an observed value is the “real” value plus an “experimental error” described by a Gaussian probability distribution. Computational studies exploring models for systems in nature are called “simulations” and are often performed on models believed to be accurate, with the goal of obtaining information that is difficult or impossible to obtain from the observation. Simulations that include a model for the scientific instruments are often labeled as “virtual experiments”. Computational studies applying statistical models to the data are called “data analysis” and typically have the goal of determining a set of model parameters that best describe the data resulted from the observation or simulation. The arguments I present in this paper apply to all of these categories.

Many scientific models are formulated in the framework of a *theory* which defines the general rules for a large class of models. An example is classical mechanics, which is a theory describing the dynamics of systems of point masses or finite-volume rigid bodies. Within the framework of classical mechanics, a model for a concrete system can be defined by a single function called a Hamiltonian. Theories play an important role in the most mature fields of science (e.g. physics) but are not essential for defining models. Younger disciplines, e.g. systems biology, construct models in a more *ad hoc* fashion without a clear underlying theory. Yet another approach is the construction of models derived from several theories in a multidisciplinary setting, e.g. in climate research. For the aspects that I discuss in this article, it does not matter if a model is developed in the context of some theory.

*Computable models* are the models that are of prime interest in computational science. A computable model is a computable function, as defined in computability theory ^11^, whose result can be compared to data from observations. Since validation requires the comparison of concrete results with observed data, one would expect that all quantitative models in science are computable models. Surprisingly, this is not the case. In fact, most mathematical models used in science are not computable.

Consider, for example, the description of the solar system in terms of classical mechanics that goes back to Isaac Newton: a set of point masses (the sun and the planets) interacting through Newton’s law of gravitation and moving according to Newton’s laws of motion. The latter are differential equations for the positions and velocities of the celestial bodies. Together with a set of parameters obtained from observation (for example, the positions and velocities of all celestial bodies at a given moment in time), these equations determine the positions and velocities at any time in the past or the future. However, they do not provide a recipe for computing the actual numbers that could be compared to observations. An additional *approximation* is needed to obtain a computable model. For the simplest case of a system of only two celestial bodies, an analytical solution of the differential equations can be obtained. This solution contains transcendental functions (sines and cosines), which are computable to any desired precision. However, when three or more celestial bodies are included in the model, no analytical solution is available and the differential equations must be approximated by finite difference equations ^12^. The development of computable approximations to the Newtonian model of celestial dynamics remains an active topic of research (see e.g. ^13^). More generally, one can consider the whole field of numerical analysis as dedicated to constructing computable approximations to non-computable mathematical models.

It may seem surprising that most mathematical models used in the most mature domains of science do not strictly speaking deserve the label “scientific”, because they cannot make predictions that are immediately comparable to observed data. The explanation is that computation was for a long time considered a menial task not worthy of the attention of a distinguished mathematician or scientist, who should concentrate on mathematical and logical reasoning. As Dowek ^14^ explains in a fascinating account of the interplay of reasoning and computation in mathematics and logic in the course of history, the important role of computation in formal reasoning has become clear only during the 20th century. While today it is generally accepted by mathematicians and logicians, most theoreticians in the natural sciences still consider computation an inferior approach to exploring scientific models, which is only used out of necessity when other techniques have failed. I suspect that this lack of interest by “real theoreticians” for the computational aspects of science might have contributed to the problems that I have outlined in the introduction. This would also explain why computational training is still largely absent from the science curricula around the world.

Scientific models can be written down in many ways: mathematical equations, diagrams, plain language, etc. The same model can be represented by different notations. For example, in principle any mathematical equation could be replaced by a verbal description. Computable models can be expressed in any Turing-complete formal language, and in particular in any of the commonly used programming languages, making them the most precise and unambiguous scientific models. The very fact that a program runs and produces results proves that the model specification is complete and unambiguous, assuming that the computing system itself (hardware, operating system, compiler, etc.) works correctly and that the programming language it is written in has clearly defined semantics (which, unfortunately, is not the case for widely used languages such as C ^15^. The utility of computation in the process of understanding and documenting science has been pointed out by Sussman and Wisdom ^16^, but is not yet widely recognized in the scientific community. A nice illustration from an engineering domain (the design of musical instruments) is given by Mairson ^17^, who designed a computable notation for describing the geometrical constructions that have been used for a few centuries to construct string instruments. His notation is meant to be both a set of instructions for a computer and a precise and unambiguous description for human readers.

A final important point about computable models is the importance of correctly identifying, understanding and documenting approximations. Scientists frequently make approximations to computational models without recognizing them as such, and therefore do not document these approximations in their publications. A good example is the use of finite-precision floating-point numbers in place of real numbers. Most scientists would consider this a technical necessity in implementing a model on a computer, and therefore an implementation detail of computational software. However, floating-point numbers have properties that differ significantly from real numbers (for example, addition and multiplication are non-associative), and the finite precision necessarily changes the results of the computations. Making such approximations explicit would also encourage the consideration of alternatives, e.g. the use of interval arithmetic. In general, any modification to a computer program that changes its results implies an approximation to the original computational model. This also includes techniques such as lossy compression of output data, which again are usually considered implementation details.

In summary, computational science involves working with computable scientific models, which are either constructed from first principles or more frequently as approximations to non-computable models. A publication describing a computational study should contain a full description of the models that were actually used in the computations. For the models derived as approximations, this means that the final approximation, though preceding steps in the derivation, should also be given in order to document the process. Computable models can be expressed unambiguously in a Turing-complete formal language. A suitable Turing-complete language should be the preferred form for publishing models.

## Tools and methods

Scientists use a variety of tools to gather observational data, explore the predictions of models and perform comparisons between them. I use the term “tool” in a general sense that includes both physical objects (e.g. microscopes, lasers, etc.) and mathematical theorems or procedures (e.g. calculus or algebra), but not mathematical axioms and definitions, which form the language of mathematics rather than its toolbox. Both computers and the software that runs on them are thus considered tools. Tools are evaluated by how well they help us in getting a job done, which leads to criteria such as precision, performance, efficiency, convenience and price. In scientific publications, the tools are described in the “Methods” section. A computational method corresponds to running one or more software tools with specific input parameters.

People using tools, not only in science, develop a *mental model* of how the tools work and what they do. Such mental models are mostly empirical and are developed by training and experience. They are personal and not formalized in any way. There is no fundamental difference in how we form mental models of a car, a microscope, and a text editor running on a computer. Our mental models are limited to the aspects of the tools that we have to know, and they do not include the tools’ inner workings or construction details. For example, to drive a car, we need to understand accelerating, braking and steering, but not the process of combustion in the engine. Similarly, we can use a microscope or a text editor with far less knowledge than it takes to design and build one. However, the domain of application and the precision that we can expect from the results are part of the mental models that scientists need to have for their tools.

While tools are indispensable for conducting science, they are not considered as part of the outputs of science, which consist of validated models. Articles documenting scientific studies describe the tools and methods that were used in the experiments or computations in order to permit readers to judge the pertinence of the conclusions drawn from the outputs. The development of new tools is also described in scientific publications because these tools are important products of the scientific research process. Nevertheless, these two aspects (tools and outputs) should be kept separate. The conclusion of a scientific study needs to be independent of a specific tool to deserve the name “scientific”. Another scientist should be able to reach the same conclusion using different tools, which is part of the requirement of reproducibility.

In computational science, the distinction between models and methods is not always very clear, because both take the form of algorithms. Some disciplines, e.g. bioinformatics, are very methods-oriented and rarely refer to models. A bioinformatician is more likely to propose a “method to predict protein folding” than a “model for protein folding”. This is partly due to differences in scientific jargon among disciplines, but it also reflects deeper issues concerning the role of computing in science. The global minimum of a knowledge-based potential for proteins is clearly a scientific model for a native structure. It is even a computable model in the sense of computability theory, in that there are known algorithms that can find the global minimum in finite time to any specified precision. However, that finite time is so long on today’s computers that the global minimum cannot be computed in practice. Bioinformaticians therefore construct heuristic methods that find structures close to the global minimum rapidly in the majority of cases. If these heuristic methods are deterministic, they should be considered approximations to the original model. This is not an option for heuristic methods that involve random choices, because they do not produce a unique result for a given input and therefore do not qualify as scientific models.

It is important to distinguish the use of randomness in heuristics from the use of probabilistic models, i.e. models that predict observable quantities as averages over probability distributions. The latter are in the same category as the global-minimum example discussed above: the numbers they predict are well-defined and computable, even though their computation is often beyond the limits of today’s computing technology. By contrast, a method such as *k*-means clustering, whose initialization step requires an arbitrary random choice, yields a different result each time it is applied, and there is no reason to attribute any meaning to the statistical distribution of these results. In fact, the distribution used in the initialization step is hardly ever documented because it is considered irrelevant. The role of such heuristics in computational science remains to be clarified.

## The double role of scientific software

The dominant role of software in our lives is the role of tools. A computer program *does* something: play videos, manage bank accounts, simulate protein dynamics, etc. A software is developed explicitly for doing something, and is evaluated by how well it performs the task. In most situations where software is used, there is a clear distinction between the software as a tool and the content that the tool works on. A video player is distinct from the movies it plays, and this distinction is visible to everyone: there is one file on the computer for each movie, and one file (or set of files) for the video player. The same video player can play many movies, and for each movie file there are multiple computer programs that can play them. The same clear distinction holds between the software that manages bank accounts and the databases that contain the actual data. However, it does *not* hold for the simulation of protein dynamics. A simulation is the computation of a prediction from a model, but there is no computer file that holds a model for protein dynamics, and another file that holds a simulation program. The model is an integral part of the simulation program. The files read by that program contain some of the *parameters* of the model (e.g. the initial structure of the protein), but not the model itself. There is no clear separation between the tool and the model it operates on.

This fusion between models and tools in computational science is problematic because models and tools have very different roles in science and are evaluated according to very different criteria. Blurring the distinction leads to a number of undesirable consequences:

**Lack of understanding:** In the theoretical sciences, researchers should know and understand in detail the models they apply. These models are shared by a research community, and formalized using a suitable standard notation to reduce ambiguity in communication. Scientists do not have the same detailed understanding of their tools. Researchers using scientific software (as opposed to those who develop it) work with an empirical mental model of that software, as explained above. When scientific models are hidden inside the software, the higher level of understanding required for them becomes very difficult to develop. As a consequence, researchers cannot make an informed decision between different models and often choose the more convenient or more efficient program, regardless of the model that it implements.

**Lack of verification:** New tools should be tested by running them on well-known models as test cases, for which they should produce exactly the same results. New models should be tested by comparing them with well-known ones, using exactly the same tools. Software that inextricably combines complex models and complex technology becomes nearly impossible to evaluate. Moreover, formal proofs can be used to validate software tools against a formal specification. But formal proofs cannot handle scientific models (they are validated against observations), and therefore they cannot handle tools with built-in models either. Tools can only be validated using formal proofs if they work on models that are external.

**Interdependence:** Models and tools should evolve independently: models are improved with the progress of science, whereas computational tools are improved following changes in computing technology, or simply by investing more efforts. When the models are part of the tools, it becomes difficult to distinguish an improved tool from an improved model. Moreover, changes to the tools for technical reasons (i.e. accelerating a computation using Graphics Processing Units (GPUs)) often require approximations to the embedded models, which tend to remain undocumented because they are not recognized as such.

All these consequences can be observed in the field of protein simulations. It is generally accepted in the protein simulation community that it is impossible to obtain the same numbers for a given system from two different simulation programs (lack of verification). Most scientists understand that this is due to no two programs implementing exactly the same model. However, few if any practitioners are able to explain how exactly these models differ (lack of understanding). It is also considered inevitable that different versions of the same program, or even two executables compiled with different compilers or compiler options, produce slightly different results for what should be the same model (interdependence).

It is important to understand the practical differences between a computable scientific model and a software tool. From the point of view of theoretical computer science, both are programs and both are expressed in a Turing-complete language. However, the model specifies just the result of a computation. The software tool defines how to perform a computation efficiently on data read from and written to permanent storage, within the constraints of a given physical computer. This requires handling aspects such as the use of resources (memory, CPUs), I/O, and possibly parallelization. A practically useful software tool also requires attention to the user interface, to file formats, and other tool-specific characteristics. In a typical scientific software tool that integrates models, the vast majority of the source codes is dedicated to these technical aspects, to the point that it can be difficult to identify the models in the source code.

It is interesting to analyze why the fusion of models and software tools is possible and how it occurred. In the cases of video players and bank account management cited above, the separation of tools and data seems evident. The tools consist of instructions for the computer, the data is ultimately just a sequence of numbers. Anyone who has written simple programs is able to see at a glance the difference between software (text files containing instructions in a programming language) and data (tables of numbers and text for the bank accounts, compressed binary files for movies). The archetype of a scientific model is a set of mathematical equations. This seems much more similar to a program than to data, all the more since most programming languages provide syntax for mathematical formulae that look similar to written maths. Moreover, as explained above, computable models actually require a Turing-complete notation. A programming language is thus a natural fit: it is very straightforward to translate a computational model into a program code. On the other hand, it is not at all straightforward to write a program that reads in a scientific model as it would read in “normal” data. So it seems that scientific models are in fact programs rather than data.

However, the distinction between “program” and “data” doesn’t stand up to scrutiny. Programs *are* data. They are stored in files, can be copied around, e-mailed, etc., just like any other piece of data. Compilers read source code files as data and transform them into executables, which is just a conversion of data into another form. The distinction between programs and data that seems so obvious to computational science practitioners is just a historical accident. The programming language Fortran ^18^, which made large-scale scientific computing possible in the late 1950s, made this distinction for practical reasons: it allowed the development of simple and efficient compilers. Lisp ^19^, another programming language developed in the late 1950s for research in artificial intelligence, made the opposite choice: a program is just a particular interpretation of a data structure. Lisp programmers routinely assemble data structures and then execute them as programs. However, early Lisp implementations were slow compared to Fortran, and thus never became popular in computational science, with the notable exception of computer algebra systems.

In the early days of computational science, a theoretician would define a model with pencil and paper, and then write a program to do a specific computation based on that model, such as computing an integral or solving a differential equation numerically. The computation on the computer simply replaced the earlier practice of manual computation. Models were published in journal articles, just like in the pre-computing era. A computer program was considered an *implementation* of the model and testing the program involved comparing its output with results from analytical manipulation of the model for suitable input values.

With the rapid increase of computational power, scientists could handle ever more complex models, and in particular models far too complex to be managed with pencil and paper. But scientific publication remained in the pencil-and-paper world for a few more decades, because electronic communication became feasible only with the rise of the Internet in the 1990s. Scientists could thus work with computational models that were too complex for publication, and as a result they stopped publishing their models. With the separation of models and programs being discouraged by the computational tools, and in the absence of any motivation to formulate computational models independently from programs for communication, the fusion of models and programs became almost inevitable.

## Software as a notation for scientific knowledge

In the previous section, I have explained the undesirable consequences of the fact that computational models are often inseparably intertwined with the software tools that work on them. There is another important problem resulting from the fusion of tools and models, which is related to the different time scales on which science and computing technology evolve at the moment. This problem could disappear in the unlikely case that progress in computing technology slows down in the future, but it currently requires immediate attention if we want to preserve the scientific heritage of the last decades.

Knowledge has a finite lifetime. Even if information storage media could be preserved forever, the meaning of the information they contain is ultimately lost because the semantic context in which it was encoded cannot be recorded. The best examples are historical written documents that nobody can read today, because the languages and writing systems used at the time have disappeared ^20^.

Written human languages are the most stable semantic contexts we have: they change on a time scale of centuries to millennia. Scientific jargon and scientific notations are even more short-lived. Journal articles written 100 years ago are difficult to understand for today’s scientists. The original writings of Galileo or Newton can be understood only by scholars specialized in the history of science. The time scale on which original publications remain understandable is a few decades. This doesn’t mean that knowledge is lost rapidly. As the original writings become less and less clear, the aspects that are recognized as particularly important are constantly reformulated in review articles, monographs, and textbooks. This is why the insights of Galileo and Newton are still accessible to today’s physicists.

Software as a notation for knowledge representation has a much shorter lifetime than scientific writing and mathematical notation. There are two approaches to understanding software: (1) studying it theoretically, by reading the source code and the documentation, and (2) observing its behavior, by running the program. Practice has shown that both approaches must be combined for a successful understanding of non-trivial software. Reading the source code permits making hypotheses about what the program does, which are then checked by running it on suitable input data. Source code remains intelligible as long as the language it is written in remains in active use. Depending on the language, this implies a time scale of a few years to at best one or two decades. Running a piece of non-trivial software without modifications is rarely possible after more than a few years. Software requires regular “maintenance” to remain usable. This maintenance consists in updating the source code and the installation procedures to adapt them to changes in the computing environment (compilers, operating systems, etc.) and in the dependencies (libraries, etc.). Maintenance is expensive and economically feasible only for widely used programs. Moreover, it generally proceeds in parallel with improvements in the models and methods implemented by the software. Today’s working version of a piece of scientific software does not necessarily reflect the models and methods that were implemented in its predecessor used a few years ago for an important computational study. Technical solutions such as the use of version control systems and archiving the exact code used for a specific scientific study can help to alleviate this problem, but they are not a panacea: they do not provide a code that works 30 years from now and implements today’s models and methods.

The consequence of the different time scales on which scientific knowledge and computing technology evolve is that we are losing scientific knowledge encoded in the form of software faster than it can be integrated into the reformulation process of science. For many computational studies performed during the last decades, it is already impossible to find the exact models and methods that were used. By applying the recommendations of the Reproducible Research movement, i.e. by publishing and archiving software and data, we can preserve the original expressions of this scientific knowledge, but not the semantic context.

This knowledge rot problem concerns not only models and methods that are embedded in scientific software, but also data stored in formats that are proprietary and thus defined by the software that reads and writes them. When the software becomes unusable, the data becomes unreadable. This aspect is much more widely recognized and there is a general consensus among experts in scientific data management that proprietary data formats are unsuitable for publishing and archiving purposes. The same attitude should be adopted with respect to models.

## Shifting the focus from tools to models

Solving the problems that I have discussed above would require most of all a shift of focus in computational science. Instead of concentrating on tools, which then subsume models and imprison data, we should focus on models and data as the primary items of interest for science. Before thinking about the question “How can I best do this computation?”, we must first consider the questions “What data and models does this computation depend on?” and “What will be the result of this computation?”

Such a shift of focus does not happen overnight. On the contrary, I would expect it to take many years, or maybe decades. In the following, I will outline some concrete steps to make it happen. First, I will discuss short-term actions that can be taken immediately and do not require profound changes to the scientific software and workflows that we use today. These actions will improve the understanding of the models implemented in scientific software, and will make it possible to discuss models in the scientific literature. I will then describe a second set of actions which require a serious research and development effort, but also offer significant benefits in return: the possibility to turn scientific models into first-class digital objects that can be published and archived, and the possibility to verify scientific software by formal proofs.

The main short-term action that must be taken is a thorough documentation of the scientific models that are implemented in a piece of software. Such documentation should explain the models in plain words and in mathematical notation, and point the reader to the relevant parts of the source code. Moreover, it should discuss how compilation and installation options and data in input files affect the models. As a guideline for deciding if a given feature belongs to the model or the tool, consider the interpretation of the results of the computation. Anything that changes these results in a way that must be understood for their interpretation is part of the model. Computational studies should cite the model documentation of the software that was used, and provide the values of all relevant compiler and installation options and input parameters.

A related short-term action is writing reference implementations of scientific models in the form of programs optimized for clarity rather than performance or flexibility. Such reference implementations are at the same time a precise documentation of the model and executable programs whose results can be used to validate the results of more complex software written to be used as a tool. Writing reference implementations, like writing better documentation, takes time, and therefore one condition for making it happen is the creation of suitable incentives.

Further useful actions can be taken to improve scientific software without introducing any profound changes. User interfaces, a category which includes command-line options and the syntax of input files, can be redesigned to clearly separate model-related information (typically model parameters) from tool-related information. A clear distinction helps users to better understand the techniques they apply. Software developers can also aim for better modularity with respect to models: the source code of the program can be restructured to concentrate model-related aspects in as few source code files as possible.

Reaping the full benefits of a separation between models and tools requires more profound changes to the structure of scientific software. Models and tools must become distinct entities, which are developed, tested and published independently. Tools read in model specifications as input data. Such an approach is technically feasible today, due to the enormous progress that computer science has made since the 1950s. Domain-specific languages can be designed for the definition of scientific models, and translated by tools based on compiler technology into efficient code for today’s and tomorrow’s computers. A significant amount of research and development remains to be done, but it is justified by the improvements in the quality of computational science that it will make possible.

The most immediate benefit is that models will become well-defined citable entities. A model that has been specified in a formal machine-readable notation can be published and cited via a Digital Object Identifier (DOI). Tools can be written to define, explore, modify and evaluate models. In particular, tools that are very similar in spirit to today’s computer algebra systems can be used to create approximations and combinations of scientific models. Theoreticians will be able to work with computable models in electronic form just like they used to work with mathematical models on paper in the past. Formal model specifications are not subject to the rapid evolution in computing technology, and can therefore be expected to be much more stable over time than today’s models embedded in software tools.

Formalized models can also play an important role in future human interfaces to science, as used for communicating results and teaching students. It is foreseeable that static publications such as today’s articles will be replaced by dynamic and interactive presentation and visualization techniques (see 21 and 22 for examples). Creating such presentations on top of executable models ensures consistency between explanations and applications. With models being digital objects with clear semantics and a stable reference through a DOI, they become accessible to content mining and bibliometric analysis. It will be possible to compile databases of models used in published studies, which can then be annotated with validation information. The output of science shown in Figure 1 will become more formalized than it is now, which is likely to improve the quality of science overall.

Another important gain in reliability can be expected from software technology. The automatic program verification methods that are currently developed (see e.g. 23 for a non-trivial practical application) will become available for scientific software ^24^. These approaches use automated proofs to verify that a program’s output conforms to its specification. These cannot be applied to today’s scientific software because it has no formal, and thus machine-readable, specification. The reason for this are the integrated models. Mathematical proof techniques cannot validate a model, because its validity is determined by comparison to observational data. However, given a formalized model, mathematical proof techniques can verify that a software tool correctly implements this model. This is probably the single most important element for improving trust in scientific software and thus computational science. However, to make this happen, a much closer collaboration of computational scientists and computer scientists would be required in the future.

### Floating-point arithmetic

The specificity of floating-point arithmetic deserves a special discussion, both because of its central role in much of scientific software and because of its reputation of being the source of intractable problems.

First of all, it is worth pointing out that floating-point arithmetic can be defined as rigorously as integer arithmetic. The IEEE 754 standard ^25^ provides a well-defined data representation at the bit level and a set of well-defined deterministic operations. Much of the mysterious behavior attributed to floating-point arithmetic is due to the fact that programmers and programming language designers reason about floating-point numbers as if they were real numbers, in particular assuming associativity for addition and multiplication. This happens partly by mistake (a lack of understanding of floating-point arithmetic), and partly out of the desire to create more opportunities for code optimization by compilers (see the discussion in 23).

Unfortunately, none of the programming languages currently popular for scientific computing define the semantics of floating-point operations precisely enough to give the programmer a full control over the result of a calculation. As a consequence, the output of any program using floating-point arithmetic depends on choices made by compiler writers. Thus a scientific model specified with precise floating-point semantics cannot be implemented correctly using today’s scientific programming languages. This situation is in fact a consequence of the attitude that I have described in the introduction: computational science is so much focused on the performance of the computations and so little on the correctness of the results that there is no incentive for language designers and implementors to improve the situation.

However, this does not mean that the actions I have described above are doomed to fail. The goal is to change the currently dominant attitudes. This should also lead to the development of programming tools that provide full control over floating-point operations. Moreover, it is not at all evident that floating-point numbers will continue to occupy a dominant role in scientific computing in the long run. Their popularity is mainly due to the at least apparent ease they offer for constructing computable approximations to the scientific models of the pre-computing era, which use real numbers to describe continuous physical quantities. It is well possible that other number representations will be used in the future. The recently proposed DEC64 format ^26^, which aims to replace both integers and floating-point numbers, shows that there is still interest in improving number handling in computer software.

## Related ideas and approaches

The problem that technical details tend to swamp the result-relevant aspects in program source code is not specific to scientific computing. Among the many software engineering approaches that aim to improve the situation, Model-Driven Engineering ^27^ is the one most similar to the approach that I have outlined. It introduces the notion of a model as the specification of what a program is supposed to do. Program generators then produce an efficient implementation. However, like all of software engineering, Model-Driven Engineering has the goal of producing better tools. The models are little more than tool specifications, and are normally not accessible to the users of the finished software.

Several scientific software packages are based on domain-specific languages (DSLs) that allow users to write down certain aspects of their problem in a notation that is more compact and familiar than a programming language. An example is the FEniCS package for solving differential equations ^28^, whose DSL provides a means to write a differential equation in a notation that is close to traditional mathematics. However, the focus is on the mathematical equations rather than on the computable model, which consists of more than just the equations (boundary conditions, meshes, etc.). The distinction between the DSL and the implementation language is made for convenience of notation, not for a separation of concerns. This characteristic is shared by the other scientific DSLs that I am aware of. These DSLs have the goal of facilitating the technically most challenging part of encoding science in a computer program, but they do so clearly in the context of tool development.

Orchard and Rice ^29^ propose an “agenda for programming language research” in computational science which addresses many of the topics discussed here from the point of view of programming language research. Their agenda represents a continuation of the DSL approach described in the last paragraph. The authors insist particularly on the separation of concerns between scientific models and software implementation details, and propose a path of evolution for existing scientific software. Their article contains many references to prior work of interest.

Murray-Rust and Murray-Rust’s “Reproducible Declaratron” ^30^ proposes and implements ideas which are similar in many respects to what I have outlined in this article. Their approach is based on a long-term effort towards making scientific documents more precise and at the same time machine-readable by adding semantic markup. For example, in the plain-text sentence “the experiment was run at a temperature of 21 degrees”, the temperature specification would be replaced by XML elements indicating the type of quantity (temperature), the value (21), and the unit (degrees Celsius), with each part having a clearly defined meaning written down in a dictionary. The “Reproducible Declaratron” adds computation to this framework, applying the principle that formulae and algorithms are data. The authors do not make an explicit distinction between computable models and computational tools. They do make the distinction between a “formula” and a “computation”, which for the examples they discuss is very similar to the model-tool distinction, but is limited to models derived from mathematical equations. An outstanding feature of their approach is that it moves formulae and computations from computational tools into scientific publications.

Finally, my own ActivePapers project ^31^ provides a framework for computational science that does a first step in the direction I advocate in this paper: it shifts the focus from doing computations to publishing computational methods and results. An ActivePaper is a publishable and citable package of data sets, including executable codes as another kind of data. Every data set in a published ActivePaper has an automatically resolvable reference. Moreover, the framework was explicitly designed to include code transformation and code generation. However, suitable domain-specific model languages and tools that work on them remain to be developed.

## Conclusion

In the preceding sections, I have explained that (1) the way we currently perform and publish computational science is unsatisfactory and that (2) we can and should improve our attitudes and technology. The situation I have described is a symptom of a lack of exchange between the natural sciences and research in computer science. Today’s computational scientists see computer science as an engineering discipline that provides them with ever increasing number crunching power. Their own training in computational techniques is usually limited to managing the practicalities of working with software tools. From the other side of the fence, computer scientists see scientific computing as almost synonymous with high-performance computing.

In the past centuries, much of the progress in science was due to an interplay between mathematics and physics in a domain of research now called “mathematical physics”. It was conducted by scientists who were at the same time application-oriented mathematicians and mathematically minded physicists. Science in the 21st century would benefit from a similar approach at the interface between computation and theoretical science. Computational scientists would discover that computers are not only convenient slaves to which they can offload laborious computations, but also tools that can improve our understanding of scientific models. Computer scientists would discover how computation plays a fundamental role in our efforts in understanding natural phenomena.
