# Supplementary material for: Computational science: shifting the focus from tools to models
Source: F1000Res. 2014 Jun 17;3:101. Originally published 2014 May 7. [Version 2] doi: 10.12688/f1000research.3978.2 (PMC4184301; doi:10.12688/f1000research.3978.2)
Supplement: Peer review history

# Peer review: Referee response for version 2

**DOI:** 10.5256/f1000research.4790.r5180

**Published:** 2014-06-30

**Authors:** Patrik Jansson, Cezar Ionescu

**Affiliations:** 1 Computer Science and Engineering, Chalmers University of Technology, Gothenburg, Sweden

The author has acted on almost all of the comments we provided and we are happy with the result. The only remaining problem is the following sentence:

*"It may seem surprising that most mathematical models used in the most mature domains of science do not strictly speaking deserve the label “scientific”, because they cannot make predictions that are immediately comparable to observed data."*

In the new context where sin and cos are explained to be computable to any desired precision, the conclusion of non-computability of "most mathematical models" no longer follows. (Data measurements are also of limited precision.) This does not make the sentence false (it is now indeed very surprising), but it probably needs reformulation.

We have read this submission. We believe that we have an appropriate level of expertise to confirm that it is of an acceptable scientific standard.

# Response

**Published:** 2014-06-30

**Authors:** Konrad Hinsen

**Affiliations:** Centre de Biophysique Moléculaire (CNRS), France

For the case of Newton's equations, solutions are computable only for the two-body problem. The two-body problem is of purely pedagogical interest today, as astronomical observations are so precise that they require taking into account at least the sun and all the planets, sometimes even the bigger moons. The computability of the two-body problem makes no practical difference: Newton's equations for the solar system do not have computable solutions for any practically relevant situation in astronomy.

More importantly, I think it is clear from my article that Newton's solar system model is merely an example. My claim that "most" mathematical models are not computable can clearly not rely on this one example. What motivates this statement is the importance of numerical analysis in science, as I explain in the immediately preceding sentence.

# Peer review: Referee response for version 2

**DOI:** 10.5256/f1000research.4790.r5205

**Published:** 2014-06-20

**Authors:** David Hestenes

**Affiliations:** 1 Professor Emeritus, Department of Physics, Arizona State University, Tempe, AZ, USA

I approve of the changes made to the article, and have no further comments.

I have read this submission. I believe that I have an appropriate level of expertise to confirm that it is of an acceptable scientific standard.

# Peer review: Referee response for version 1

**DOI:** 10.5256/f1000research.4263.r4684

**Published:** 2014-05-27

**Authors:** Patrik Jansson, Cezar Ionescu

**Affiliations:** 1 Computer Science and Engineering, Chalmers University of Technology, Gothenburg, Sweden

This position paper addresses the current state of computational science (the term is not defined, but appears to mean the use of computational models as an essential means of scientific investigation).  As the author says, "the central question in computational science is: why should we trust the results of a non-trivial computation?".  At the moment, the answer appears to be: "we probably shouldn't", clearly an unsatisfactory state of affairs.

This is a point which has been made before, and the paper references a number of previous attempts to come to grips with it. The author's own approach is based on the introduction of an interesting conceptual distinction: between models and tools.  It is then argued that many of the problems in today's computational science arise because of "blurring the distinction".

That the distinction is not unproblematic is illustrated by the following difficulty. On page 4, tools are defined to be "both physical objects and mathematical theorems or procedures (e.g. calculus or algebra)".  Later, we read: "the conclusion of a scientific study needs to be independent of a specific tool to deserve the name 'scientific'". On the previous page, however, we have the example of a description of the solar system as a set of differential equations. This seems to be crucially dependent on calculus. Is it therefore not scientific?

As this example shows, the paper is sure to provide food for thought and for debate. We find that it does contribute to highlighting and clarifying the problems we encounter in computational science, and as such it deserves to be published and to reach a wider audience.

There is one error which must be corrected before that, however: namely, on page 3, where the functions "sin" and "cos" are said to be uncomputable, by virtue of being transcendent. In fact, it is well known that they are computable (as are all functions that can be expressed by power series, which is one of the examples in Turing's 1936 paper). If the author has a different notion of computability in mind, then it should be stated explicitly. (See the labels [Approx0], [Approx1] and [Approx2] below for some more details.)

Another somewhat strange statement (but not an error) is that "heuristic methods that involve random choices [...] do not produce a unique result for a given input and therefore do not qualify as scientific models".  One would think that they do, in fact, produce a unique result: namely a probability distribution, and that computer simulations can be used to sample this distribution to whatever precision necessary.

There are, additionally, a number of infelicities which might be addressed before publication, such anthropomorphic expressions ("computers help with collecting..., evaluating, ..., and communicating" instead of "computers are used for collecting ..., evaluating, ..., and communicating"), or the less-than-helpful figure 1, which does not seem to bring anything over its one line description in the caption.

**Some detailed comments:**

- Abstract: "As a consequence, these crucial pieces of information have disappeared from the scientific record." We suggest some reformulation. It is not that they have first been there and later "disappeared" but rather they were absent all along and now (with more complexity developing) they are increasingly difficult (or impossible) to generate from what is in "the scientific record".
- [Approx0] "But this solution contains transcendental functions (sines and cosines), which are not computable and therefore must be replaced by computable approximations, e.g. power-series expansions." Reformulate: perhaps move the text from [Approx2] up and reformulate.  "... there are known algorithms that can find ... in finite time to any specified precision."
- [Approx1] "they cannot make predictions that are immediately comparable to observed data": All observed data is of finite precision so it can be claimed that comparing model results to   observed data is often (but not always) feasible.
- "The very fact that a program runs and produces results proves that   the model specification is complete and unambiguous, ..." The semantics of some languages (like C) is ambiguous (non-deterministic).
- "... the importance of correctly identifying, understanding and documenting approximations." We strongly agree with this part and think it would be worth pointing to interval arithmetic as one way to handle this problem.
- [Approx2] "It is even a computable model in the sense of computability theory, in that there are known algorithms that can find the global minimum in finite time to any specified precision."   Note that this looks inconsistent within the paper: earlier (at [Approx0]) you claim that sin and cos are not computable, but with this definition they are.
- It would be preferable to skip "of course" here: "This doesn’t mean of course that knowledge is lost rapidly." := "This doesn’t mean that knowledge is lost rapidly."
- "tools that very similar in spirit" := "tools that are very similar in spirit".

We have read this submission. We believe that we have an appropriate level of expertise to confirm that it is of an acceptable scientific standard.

# Response

**Published:** 2014-06-05

**Authors:** Konrad Hinsen

**Affiliations:** Centre de Biophysique Moléculaire (CNRS), France

The referees raise some interesting points, which I hope to have addressed in the revised version of my article.

Concerning the use of calculus in defining mathematical models through differential equations, it is important to distinguish between axioms and definitions on one hand, and theorems and their applications on the other hand. I have added a short sentence to make this distinction explicit. Theorems and procedures are tools, and scientific models should indeed not depend on them. Axioms and definitions are simply the semantic context for mathematical equations, i.e. the language in which mathematical models are written. Newton's equations are based on the concept of a derivative, but do not rely on any particular theorem or technique from calculus.

The referees are right about the computability of sines and cosines. I have corrected this statement in the revision.

I have also added a paragraph at the end of the "Tools and methods" section to clarify the distinction between probabilistic models and heuristics involving arbitrary or random choices, which wasn't as carefully explained as it should have been.

Minor points that I have addressed in the revision are:

- the ambiguity of languages like C
- interval arithmetic as an alternative to floating-point arithmetic
- typos and stylistic issues

# Peer review: Referee response for version 1

**DOI:** 10.5256/f1000research.4263.r4793

**Published:** 2014-05-16

**Authors:** David Hestenes

**Affiliations:** 1 Professor Emeritus, Department of Physics, Arizona State University, Tempe, AZ, USA

This is an exceptional paper in many ways. First, it is extremely well written and well organized. Second, it deals with an important, complex subject, providing a careful, novel analysis and parsing of terms. Though the subject cuts across many scientific domains, the author demonstrates clear competence in each. Finally, I think Hinsen is right in advocating primacy of models over tools (as he has so carefully defined them).

I have read this submission. I believe that I have an appropriate level of expertise to confirm that it is of an acceptable scientific standard.
